# *Methylosinus trichosporium* OB3b bioaugmentation unleashes polyhydroxybutyrate-accumulating potential in waste-activated sludge

**DOI:** 10.1186/s12934-024-02442-w

**Published:** 2024-05-31

**Authors:** Hyerim Eam, Dayoung Ko, Changsoo Lee, Jaewook Myung

**Affiliations:** 1 Department of Civil and Environmental Engineering, KAIST, Daejeon, 34141 Republic of Korea; 2 Department of Civil, Urban, Earth, and Environmental Engineering, UNIST, Ulsan, 44919 Republic of Korea

**Keywords:** Type II methanotrophs, Activated sludge, Polyhydroxybutyrate (PHB), Waste resource recovery

## Abstract

**Background:**

Wastewater treatment plants contribute approximately 6% of anthropogenic methane emissions. Methanotrophs, capable of converting methane into polyhydroxybutyrate (PHB), offer a promising solution for utilizing methane as a carbon source, using activated sludge as a seed culture for PHB production. However, maintaining and enriching PHB-accumulating methanotrophic communities poses challenges.

**Results:**

This study investigated the potential of *Methylosinus trichosporium* OB3b to bioaugment PHB-accumulating methanotrophic consortium within activated sludge to enhance PHB production. Waste-activated sludges with varying ratios of *M. trichosporium* OB3b (1:0, 1:1, 1:4, and 0:1) were cultivated. The results revealed substantial growth and methane consumption in waste-activated sludge with *M. trichosporium* OB3b-amended cultures, particularly in a 1:1 ratio. Enhanced PHB accumulation, reaching 37.1% in the same ratio culture, indicates the dominance of Type II methanotrophs. Quantification of methanotrophs by digital polymerase chain reaction showed gradual increases in Type II methanotrophs, correlating with increased PHB production. However, while initial bioaugmentation of *M. trichosporium* OB3b was observed, its presence decreased in subsequent cycles, indicating the dominance of other Type II methanotrophs. Microbial community analysis highlighted the successful enrichment of Type II methanotrophs-dominated cultures due to the addition of *M. trichosporium* OB3b, outcompeting Type I methanotrophs. *Methylocystis* and *Methylophilus* spp. were the most abundant in *M. trichosporium* OB3b-amended cultures.

**Conclusions:**

Bioaugmentation strategies, leveraging *M. trichosporium* OB3b could significantly enhance PHB production and foster the enrichment of PHB-accumulating methanotrophs in activated sludge. These findings contribute to integrating PHB production in wastewater treatment plants, providing a sustainable solution for resource recovery.

**Graphical abstract:**

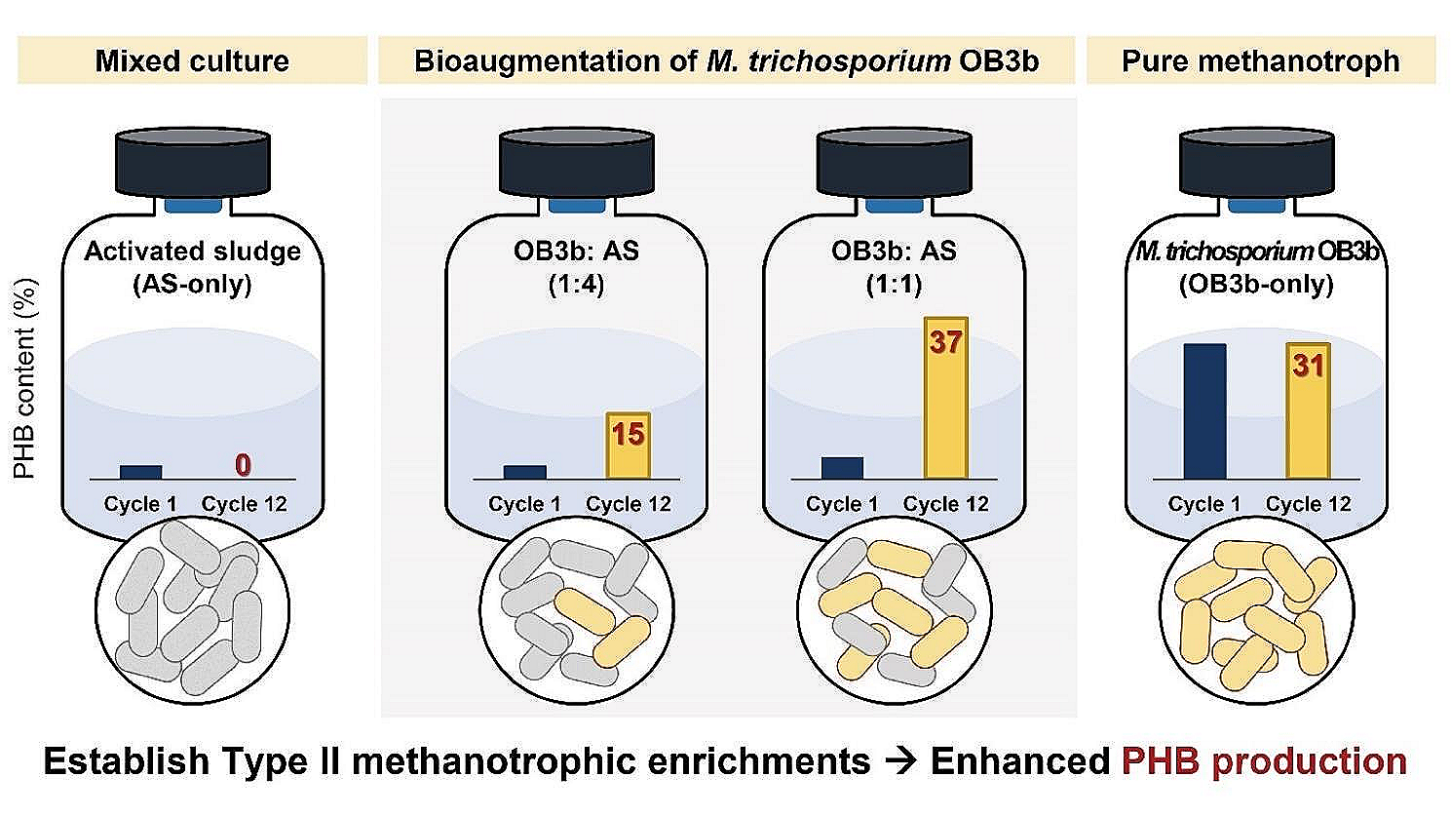

**Supplementary Information:**

The online version contains supplementary material available at 10.1186/s12934-024-02442-w.

## Background

### Methane emissions from wastewater treatment plants

Methane (CH_4_) stands out as a significant greenhouse gas, contributing to annual emissions of 576 Tg CH_4_ [1]. Anthropogenic methane sources, including agriculture, solid waste, coal mining, and wastewater treatment plants, collectively account for up to 60% of global methane emissions. Among these sources, the wastewater treatment plant sector is particularly notable, contributing 5–8% of methane emissions, followed by livestock (31%), oil and gas (26%), landfills (14%), and coal mining (11%) [2]. In the United States, centralized wastewater treatment plants are estimated to produce approximately 10.9 ± 7.0 MMT CO_2_-eq methane annually, with 72% attributed to plants equipped with anaerobic digestion [3].

Recent studies have explored diverse methodologies for valorizing methane into high-value-added products, such as heat and power generation, biopolymer production (e.g., polyhydroxybutyrate), and biofuel synthesis [4–6]. Incorporating polyhydroxybutyrate (PHB) production into wastewater treatment plants has emerged as a cost-effective alternative for methane valorization [7, 8]. PHB, a biopolymer fully degradable in natural environments, including soil, oceans, and landfills [9], presents a sustainable avenue. The substantial activation energy of C-H bonds (439 kJ/mol) in methane presents a formidable barrier to chemical oxidation processes [10]. Leveraging methanotrophs as biological catalysts offers a promising solution to reduce high energy requirements and convert methane into PHB.

### PHB production in methanotrophs

Methanotrophs, a subset of methylotrophs, utilize methane as a carbon and energy source for cell growth and biosynthesis [11]. These bacteria are classified into three phyla: Gammaproteobacteria, Alphaproteobacteria, and Verrucomicrobia [12–14]. Within proteobacteria, methanotrophs are further categorized into Type I and Type II based on the assimilation pathway of formaldehyde. Type I methanotrophs use the ribulose monophosphate (RuMP) cycle, whereas Type II methanotrophs use the serine cycle or the ethylmalonyl-CoA cycle (EMCP) [15–17]. Type II methanotrophs are notable for their production of PHB, through the serine pathway.

Unlike Type I methanotrophs, which have received some attention regarding PHB production, the majority of studies have focused on Type II methanotrophs [18, 19]. Notably, Type II methanotrophs, including *Methylocystis*, *Methylosinus*, *Methylocapsa*, and *Methylocella*, can yield substantial amounts of PHB [20], making them more efficient candidates for PHB production compared to Type I methanotrophs.

In the serine pathway, methane is ultimately condensed into acetyl-CoA, which enters two distinctive pathways based on conditions: the PHB cycle and the tricarboxylic acid (TCA) cycle. The PHB cycle is initiated with acetyl-CoA under nutrient-limited, typically nitrogen-restricted, conditions [4]. The TCA cycle operates under nutrient-sufficient conditions to derive energy sources. Key enzymes in the methanotrophic PHB cycle include beta-ketothiolase (*pha*A), acetoacetyl-CoA reductase (*pha*B), and PHB polymerase (*pha*C). During PHB synthesis, acetyl-CoA is condensed to acetoacetyl-CoA by *pha*A, subsequently reduced to (R)-3-hydroxybutyryl-CoA by *pha*B. Finally, *pha*C converts this precursor to PHB [9, 19].

PHB production process involves two stages: cell growth and PHB accumulation [21]. In the first stage, rapid methanotroph proliferation occurs under nutrient-rich conditions, generating ample active biomass. The primary goal of this stage is to achieve a high concentration of active biomass to maximize PHB production. Subsequently, in the production stage, PHB granules are synthesized within cells under nitrogen-limited conditions, impeding cellular proliferation. Optimizing nutritional and environmental conditions enhances PHB production efficiency. Nevertheless, the selection of proficient PHB-producing methanotrophs characterized by elevated biomass concentration, PHB content, and expedited growth rates stands as a pivotal determinant in this process [22]. In addition, contamination resistance and adaptability are crucial indicators for industrial-scale PHB production with methanotrophs.

### Selection of Type II methanotrophic consortium

The production of PHB is heavily reliant on the enrichments of Type II methanotrophs, which possess the capability to synthesize intracellular PHB. Integrating PHB production seamlessly into wastewater treatment plants and harnessing waste resources such as biogas can be achieved by utilizing activated sludge to enrich cultures dominated by Type II methanotrophs. This approach eliminates the sterilization costs associated with operating pure methanotroph bioreactors [22, 23]. The inclusion of mixed consortia, comprising methylotrophs and heterotrophs, may stimulate the microbial growth of methanotrophs either by consuming toxic metabolites (e.g., methanol and formate) or providing essential byproducts (e.g., quinone, pyridoxine, and vitamin B_12_) [24, 25]. However, constructing stable and effective microbial communities for PHB production, particularly with Type II methanotrophic bacteria, remains a challenge.

Various approaches have been explored to adjust environmental and nutritional factors (e.g., pH, methane concentration, nitrogen source, and copper) for selecting Type II methanotrophs in activated sludge. Although altering methane concentrations affects microbial population structure, it often results in the dominance of Type I methanotrophs over Type II methanotrophs [26]. Nitrogen-selection methods, such as using N_2_ gas with low O_2_ concentration, have been employed to form Type II methanotrophic enrichments. However, slow growth has been observed compared to other nitrogen sources (i.e., ammonium and nitrate) [27, 28]. Efficient PHB production requires faster growth, high biomass concentration, and elevated PHB content. Strategies have been employed to counter ammonium inhibition and prevent the invasion of Type I methanotrophs, leveraging the higher resistance of Type II methanotrophs to ammonium toxicity under elevated concentrations [29–31]. Despite the progress, selecting Type II methanotrophs still demands a considerable timeframe and may result in an unstable microbial community structure.

This study aims to assess the potential of *M. trichosporium* OB3b in activated sludge for bioaugmenting Type II methanotrophs in a mixed culture, with a focus on PHB production. This approach aims to establish robust Type II methanotrophic enrichments and expedite the start-up time. The presence of methanotrophs was confirmed by monitoring microbial growth and gas consumption (i.e., methane and oxygen). Digital quantitative polymerase chain reaction was employed to track the bioaugmentation of Type II methanotrophs and *M. trichosporium* OB3b. Microbial community composition was analyzed through 16S rRNA gene amplicon sequencing to identify accompanying methylotrophs and heterotrophs. Quantitative analysis of PHB was performed by gas chromatography equipped with a flame ionization detector to demonstrate the capability of PHB production. This approach holds promise for implementation in wastewater treatment plants to enhance PHB production for waste resource recovery, encompassing methane and activated sludge. Consequently, it could represent a definitive strategy for improving the resilience and stability of obtaining Type II methanotrophic enrichment.

## Methods

### Bacterial strain and culture medium

Activated sludge samples were collected from the second sediment aerobic basin located at the Daejeon wastewater treatment plant in Republic of Korea (36.38179°N, 127.40677°E). Upon immediate transfer to the laboratory, the samples were filtered with a 100 μm cell strainer to remove large materials. *Methylosinus trichosporium* OB3b, obtained from Prof. Sukhwan Yoon’s lab at KAIST (Daejeon, Republic of Korea), was precultured before commencing the experiments.

For cultivation, the ammonia mineral salt medium (AMS) was chosen due to its superior ability to accumulate polyhydroxybutyrate (PHB) compared to nitrate mineral salt medium (NMS) [32–34]. The AMS medium contained (per 1 L): 1 g of MgSO_4_.7H_2_O, 0.2 g of CaCl_2 ·_ 2H_2_O, 0.1 mL of a 3.8% (w/v) Fe(III)-EDTA solution, 0.5 mL of a 0.1% (w/v) Na_2_MoO_4_·2H_2_O solution, and 1 mL of a trace element solution. The trace element solution contained (per L): 0.5 g of FeSO4 ·7H_2_O, 0.4 g of ZnSO_4_·7H_2_O, 0.02 g of MnCl_2_·7H_2_O, 0.05 g of CoCl_2_· 6H_2_O, 0.01 g of NiCl_2_· 6H_2_O, 0.015 g of H_3_BO_3_ and 0.25 g of EDTA [35].

In 160 mL serum bottles, 43 mL of AMS medium was distributed and sealed with butyl rubbers and aluminum caps. After autoclaving, the media were supplemented with 0.5 mL of 10x vitamin stock, 0.5 mL of 0.4 M of phosphate buffer solution, and 1 mL of 0.5 mM CuCl_2_·2H_2_O solution. The 10x vitamin stock contained (per 1 L): 20 mg of biotin, 20 mg of folic acid, 50 mg of thiamine HCl, 50 mg of Ca pantothenate, 1 mg of vitamin B12, 50 mg of riboflavin, and 50 mg of nicotinamide. The 10x vitamin stock was filter-sterilized using a 0.22-µm syringe filter (Advantec, cellulose-acetate membrane). The phosphate buffer solution, containing 13 g of KH_2_PO_4_ and 31 g of Na_2_HPO_4_·7H_2_O per 500 mL, was used for pH adjustment to 6.8.

### Methanotrophic enrichments and PHB accumulation

**Fig. 1:**
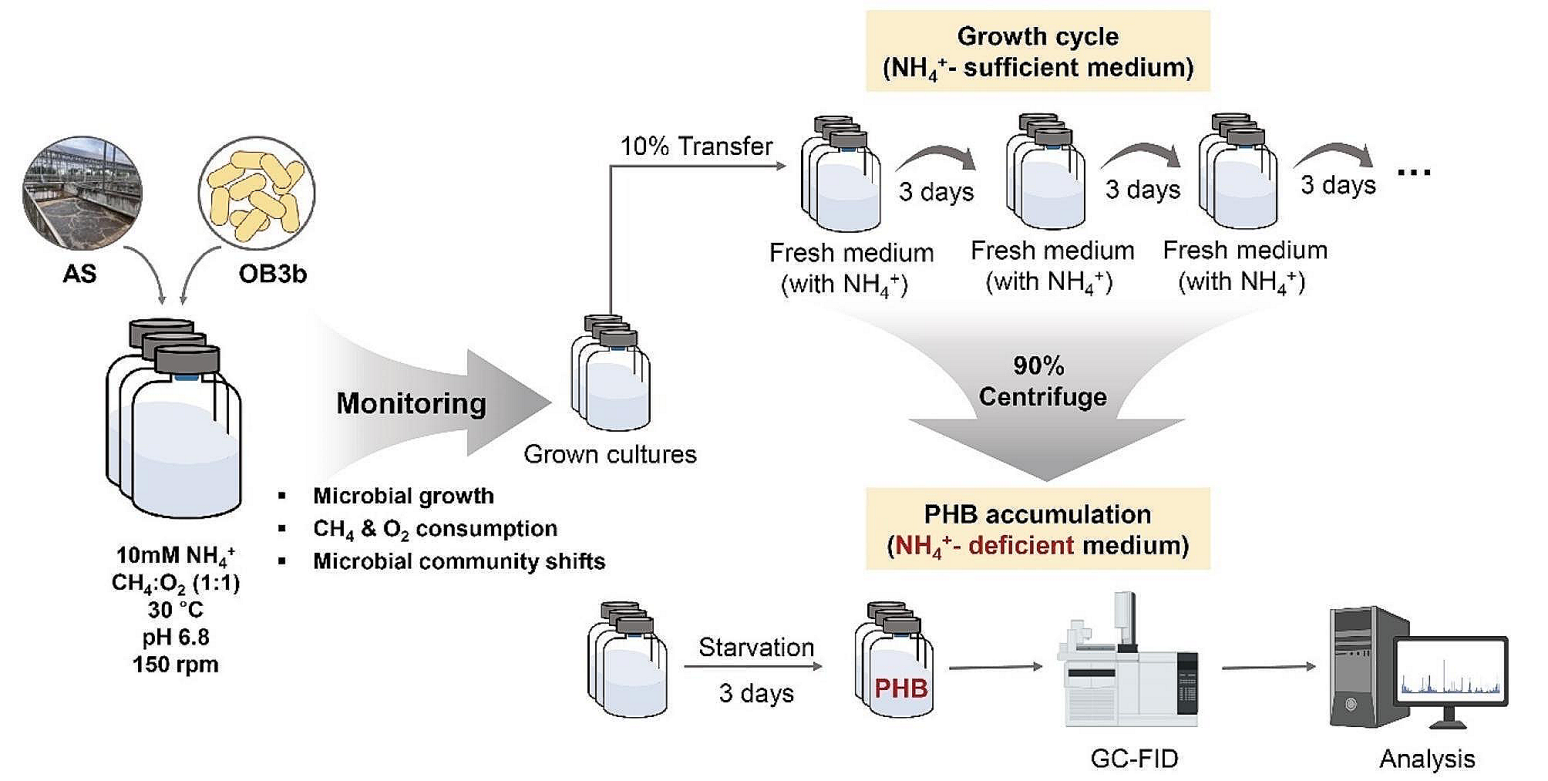
Schematic diagram of growth cycle and PHB accumulation for methanotrophic mixed cultures

*M. trichosporium* OB3b (referred to as OB3b hereafter), initially with an optical density of 0.2, was supplemented with activated sludge (referred to as AS) in serum bottles based on a biomass ratio. The inoculation ratios of OB3b to AS (w/w) were 1:0, 1:1, 1:4, and 0:1. A pure culture of *M. trichosporium* OB3b was used as a control to evaluate PHB production and methanotrophic activity (refer to Fig. 1 for an experiment schematic). These pure cultures were not subjected to repetitive cycles to minimize the risk of contamination.

Fresh AMS medium (45 mL) was inoculated with 5 mL of activated sludge with and without OB3b. For continuous growth, 10% of the previously grown culture was transferred into fresh AMS medium every 3 d to enrich the Type II methanotrophic culture. After the growth cycle, the residual culture was centrifuged at 3,500 rpm for 25 min to form a pellet. The pellet was resuspended in 5 mL of PHB medium and then injected into 45 mL of PHB medium. The PHB medium contained the same components as AMS medium but lacked ammonium. The PHB accumulation cycle was performed under nitrogen-depleted conditions for 3 d. CH_4_ and O_2_ (99.999%, Cheil Gas Industry Co., Republic of Korea) were supplied to the 110 mL of headspace and maintained at a 1:1 (v/v) ratio. All cultures were conducted in triplicate, agitated horizontally at 150 rpm at 30 ℃ in a shaking incubator.

### Analytical method

CH_4_ and O_2_ concentrations were analyzed using gas chromatography (GC) (Agilent 8890, USA) equipped with Porapak Q and MolSieve 5 A columns and a thermal conductivity detector (TCD). The injector temperature was set at 200 ℃, with argon gas serving as the carrier gas. The oven temperature program follows as: 50 ℃ for 2 min, and 150 ℃ for 2 min. For gas monitoring, 150 µL of gas in the headspace was sampled by a Hamilton-gas-tight syringe and directly injected into the GC.

Microbial growth was monitored by measuring the optical density at 600 nm by a UV-Vis spectrophotometer (Thermo Scientific, Genesys-180, USA).

### PHB quantification

The PHB content was measured by GC following established protocols [36]. After the PHB accumulation cycle, all liquid cultures were harvested, centrifuged at 3,500 rpm for 25 min, and the pelletized cells were freeze-dried, weighed, and transferred into 12 mL glass vials. Each sample underwent cell lysis, with the addition of 2 mL chloroform and 2 mL acidified methanol containing 3% sulfuric acid and benzoic acid (0.25 mg/ mL methanol). Benzoic acid and 3-hydroxybutyric acid salt (Sigma-Aldrich) were used as internal and external standards, respectively. Sealed with Teflon-lined plastic caps, all samples were heated at 100 ℃ for 3.5 h and then cooled to room temperature. To separate the organic and aqueous phases, 1 mL of deionized water was added to each sample, followed by vortexing for 30 s. The organic phase was extracted and analyzed by GC (Agilent 8890, USA) equipped with an HP-5 column and flame ionization detector (FID). The oven temperature program follows as: 160 °C for 4 min, 200 °C for 4 min, and 275 °C for 6 min.

### Digital polymeric chain reaction and microbial community analysis

Cell suspensions were collected from cycle 1, cycle 4, and cycle 12 after 72 h of growth incubation to monitor microbial community shifts using digital PCR (dPCR) and 16S rRNA gene amplicon sequencing. DNA was extracted using the DNeasy PowerSoil Pro kit (Qiagen, Hilden, Germany) following the manufacturer’s protocol. Copy concentrations of 16S rRNA bacteria, Type II methanotrophs, and *M. trichosporium* OB3b were quantified by dPCR, employing specific target primers (Table S1). dPCR reaction mixtures were prepared using the QIAcuity EvaGreen PCR kit (Qiagen, Hilden, Germany), as per the manufacturer’s protocol and loaded onto a QIAcuity Nanoplate 26 K 24-well. The reactions were analyzed in a QIAcuity ONE 2-Plex system (Qiagen, Hilden, Germany) using the following thermal cycling program: an initial heat activation step at 95 ℃ for 2 min, 40 cycles of amplification (denaturation at 95 ℃ for 15 s, annealing at 55 ℃ (for total bacteria) and 58 ℃ (for Type II methanotrophs and *M. trichosporium* OB3b) for 15 s, final extension at 72 ℃ for 15 s), and a cooling step at 40 ℃ for 5 min [37].

To assess the microbial composition of the cultures, the V3-V4 region of the 16S rRNA gene was amplified and sequenced on the Illumina Miseq platform (Macrogen Inc., Seoul, Republic of Korea).

## Results and discussions

### Growth characteristics of *M. trichosporium* OB3b-amended cultures

**Fig. 2:**
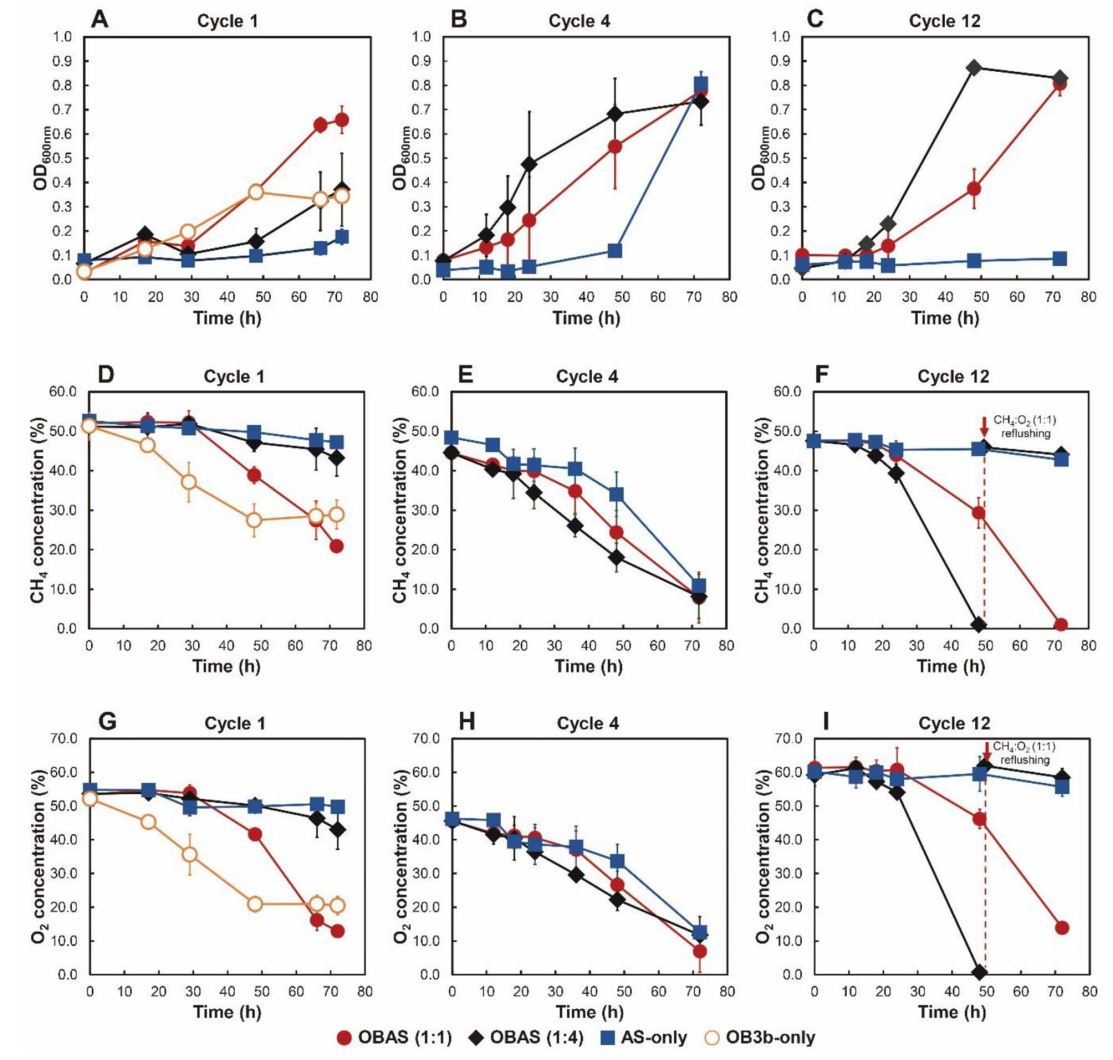
Microbial growth curves, CH_4_, and O_2_ consumption were monitored at Cycle 1 (**A, D, G**), Cycle 4 (**B, E, H**), and Cycle 12 (**C, F, I**) in OBAS and AS-only cultures

Activated sludges with *M. trichosporium* OB3b (OBAS) were incubated at varying inoculation ratios. Biotic controls, consisting of activated sludge (AS-only) and pure *M. trichosporium* OB3b (OB3b-only), were employed to assess methanotrophic activity and PHB synthesis capabilities relative to *M. trichosporium* OB3b-amended cultures (OBAS (1:4), and OBAS (1:1)). Optical density at 600 nm was used to monitor the microbial growth. Microbial growth was monitored using optical density at 600 nm, and cycles 1, 4, and 12 were analyzed for microbial growth, CH_4_ consumption, and O_2_ consumption (Fig. 2).

In Cycle 1, OBAS cultures, particularly OBAS (1:1), exhibited higher optical density compared to OB3b-only cultures, reaching a final optical density of 0.659 ± 0.056. Approximately 31% of CH_4_ and 40% of O_2_ were consumed within 72 h in OBAS (1:1) cultures, with a CH_4_ to O_2_ consumption ratio of 1.29, slightly lower than that observed in previous studies [19, 38–40]. AS-only and OBAS (1:4) cultures displayed lower optical densities of 0.371 ± 0.150, and 0.176 ± 0.031, respectively. Both cultures experienced a prolonged lag phase (within 50 h) and consumed about 8% and 11% of CH_4_, respectively. This suggests that AS-only and OBAS (1:4) cultures did not result in methanotrophic enrichments due to their lack of adaptation to the presence of excess methane.

In Cycle 4, initially injected CH_4_ was completely consumed in all cultures, accompanied with oxygen depletion. Compared to Cycle 1, faster growth and CH_4_ consumption were observed in OBAS (1:4) and AS-only cultures, indicating the enrichment of methanotrophs. However, AS-only cultures exhibited poor growth in Cycle 12, suggesting lower methanotrophic activity and a smaller portion of methanotrophs in the microbial community. This result indicated that the enrichment of Type II methanotrophs in AS-only cultures may not have been successful in maintaining the robust PHB-accumulating methanotrophic consortia over successive cycles. The optical density of OBAS cultures (1:1, and 1:4) in Cycle 12 increased to 0.807 ± 0.049, and 0.829 ± 0.031, respectively. Lag phases of each culture were within 18 h, reaching an exponential phase faster than Cycle 1. Notably, CH_4_ and O_2_ in OBAS (1:4) were completely consumed within 48 h, and cultures were refilled with methane and oxygen mixtures (1:1 v/v). No methane oxidation was observed after refilling. The methane-to-oxygen consumption ratio of OBAS (1:1, and 1:4) was calculated as 1.02 and 1.28, respectively. Previous studies reported that 1.5–2.0 moles of oxygen were required to oxidize 1 mol of methane in methanotrophic enrichments and *M. trichosporium* OB3b [36, 38, 39, 41]. Significant amounts of CH_4_ and O_2_ consumption were observed in OBAS cultures, even exceeding the levels seen in OB3b-only cultures.

### PHB accumulation of *M. trichosporium* OB3b-amended cultures

**Fig. 3:**
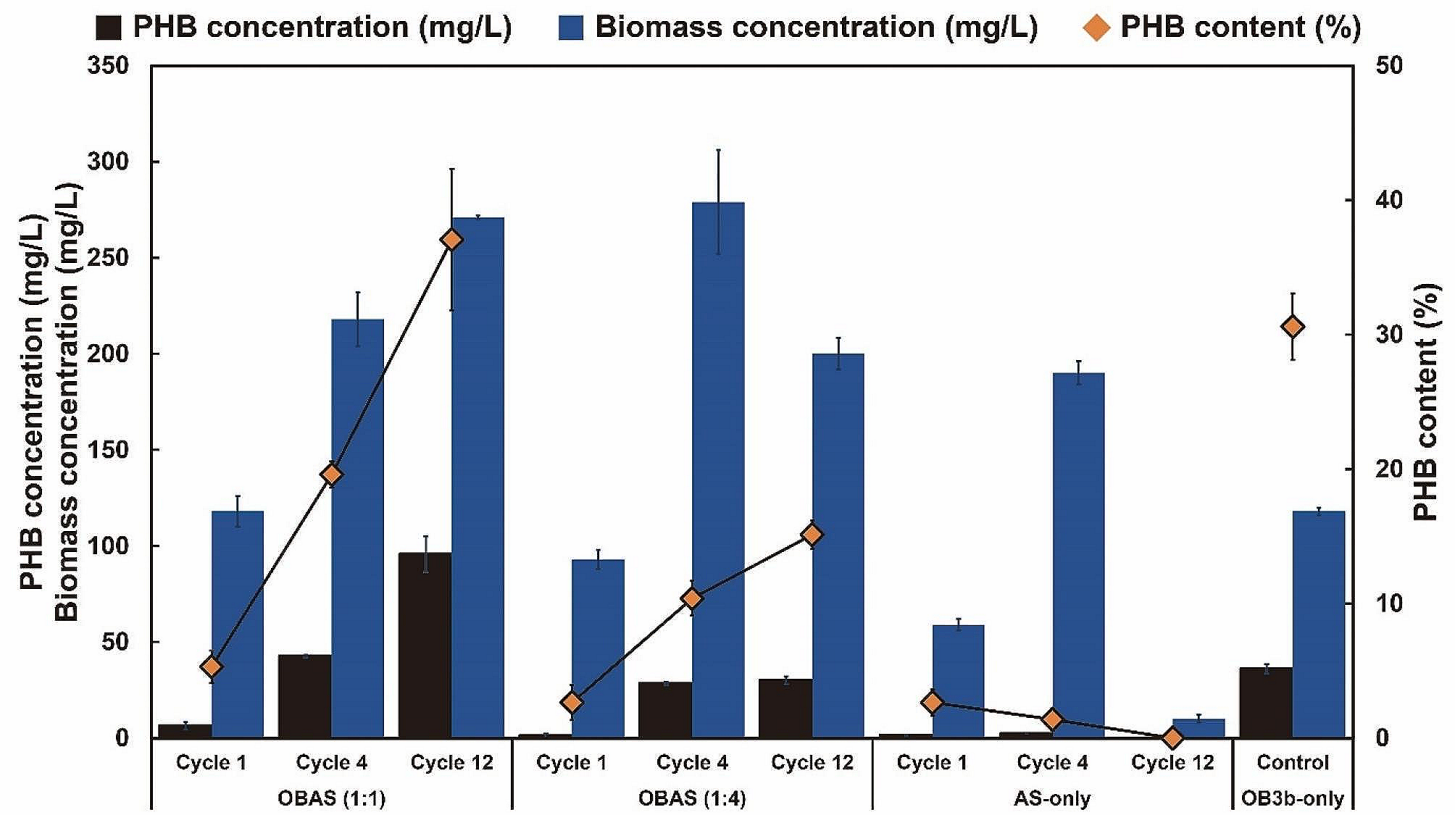
PHB concentration (mg/L), biomass concentration (mg/L), and PHB content (%) in OBAS, AS-only cultures, and OB3b-only cultures

PHB accumulation tests were conducted over a 72 h period following the growth cycle to evaluate the PHB production capability. Cells harvested during this period were transferred into a nitrogen-depleted medium to induce PHB accumulation in the presence of excess methane. PHB content was analyzed at cycles 1,4, and 12 (Fig. 3).

In the case of AS-only culture, no PHB accumulation was observed throughout all cycles, suggesting that Type II methanotrophs did not dominate in the enriched cultures. Conversely, Type I methanotrophs may have dominated, as evidenced by methane consumption in Sect. 3.1. The prevalence of Type II methanotrophs is crucial for effective PHB production. PHB contents in OBAS (1:1) cultures significantly and consistently increased from 5.3 ± 1.2% to 37.1 ± 5.3%, alongside an augmented biomass concentration. This suggests that the dominance of Type II methanotrophs, including *M. trichosporium* OB3b, in OBAS (1:1) cultures substantially occurred. OBAS (1:4) cultures accumulated up to 6.4 ± 1.8% (Cycle 1), and 15.1 ± 1.0% (Cycle 12) of PHB, with higher contents observed as the number of cycles increased. Notably, the addition of *M. trichosporium* OB3b had a significant impact on PHB content, as evidenced by the observed higher PHB contents. The maximum PHB content in OBAS (1:1) was comparable to the mixed methanotrophic consortia in previous studies, where *Methylocystis*-dominated cultures achieved up to 39% of PHB accumulation [36]. Similarly, PHB contents of 37.8% and 40% were recorded in mixed methanotrophic consortia [33, 42]. In comparison to OB3b-only cultures, higher PHB contents were obtained in OB3b-amended cultures, indicating the synergistic effect of mixed methanotrophic consortia [43].

In the context of PHB production, biomass concentration, and PHB content were closely related, affecting the PHB concentration. The concentration of PHB in cells, determined through the analysis of cell dry weight and PHB content, revealed that OBAS (1:1) cultures exhibited the most substantial biomass and PHB concentrations, amounting to 271 ± 1.0 mg/L and 95.7 ± 9.2 mg/L, respectively (Fig. 3). These values were 2.30 and 2.65 times higher than those of OB3b-only cultures, respectively, implying that non-methanotrophs in mixed methanotrophic consortia might support their microbial growth. Similar increasing trends were observed in OB3b-amended cultures concerning PHB concentration, biomass concentration, and PHB content. Consequently, the addition of *M. trichosporium* OB3B significantly enhanced the capability of PHB production (Fig. 3).

### Quantification of Type II methanotrophs and *M. trichosporium* OB3b

To investigate the dominance of Type II methanotrophs and *M. trichosporium* OB3b in all cultures, dPCR was employed to quantify the microbial population. Copy concentrations of the bacterial 16S rRNA gene were measured to determine the ratio of Type II methanotrophs and *M. trichosporium* OB3b in the enrichments, with Type II methanotrophs representing PHB accumulating methanotrophs. 16S rRNA copy concentration of Type II methanotrophs referred as Type II methanotrophs, 16S rRNA copy concentration of bacteria referred as total bacteria, and *pmoA* gene copy concentration of *M. trichosporium* OB3b referred as *M. trichosporium* OB3b in this section.

**Fig. 4:**
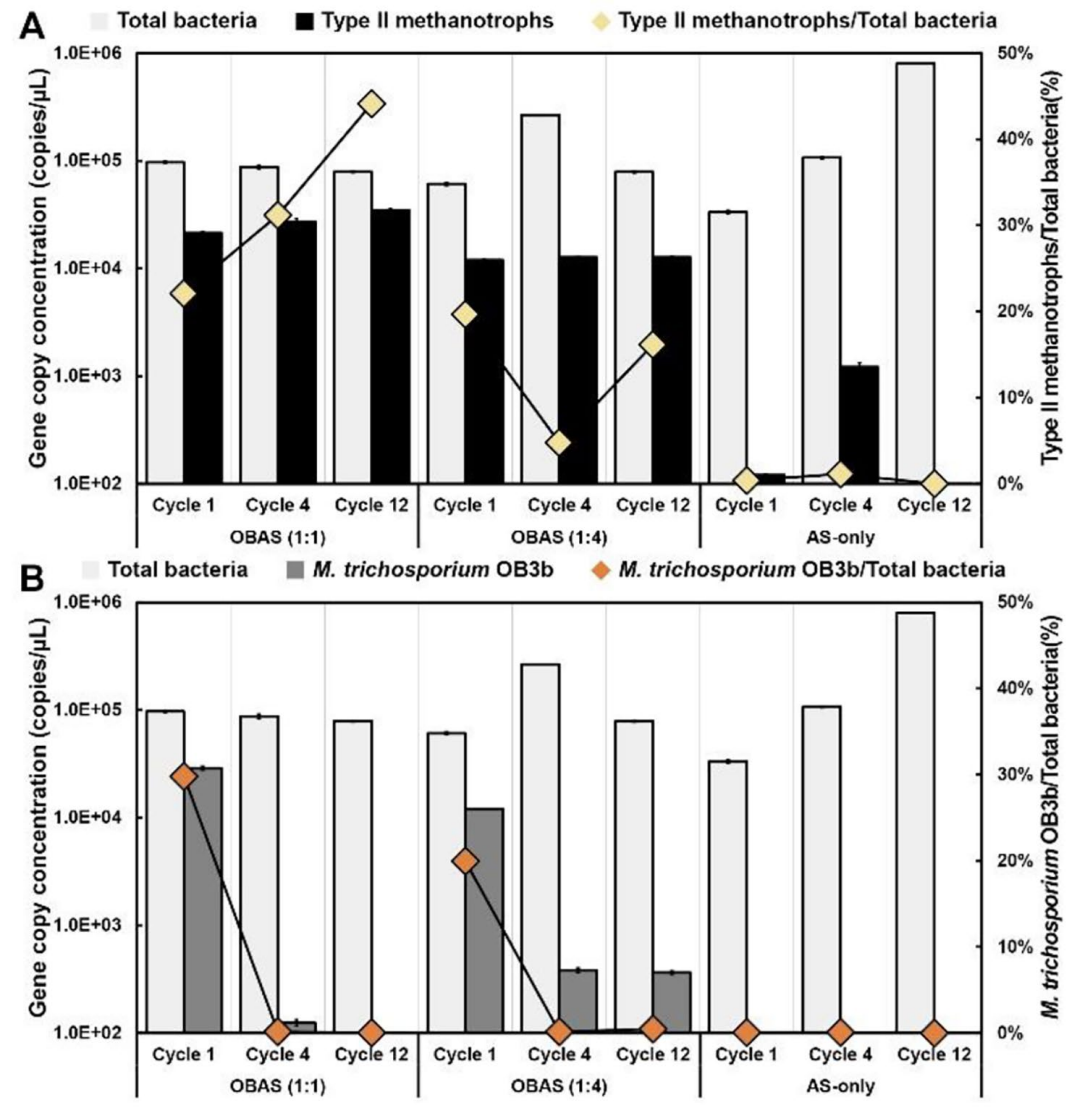
The gene copy concentrations of total bacteria, Type II methanotrophs (**A**), and *M. trichosporium* OB3b (**B**) in OBAS and AS-only cultures. The percentage of type II methanotrophs (**A**) and *M. trichosporium* OB3b (**B**) were monitored. Below 100 copies/µL was considered a limit of detection (LOD)

The initial 16S rRNA gene copy concentration of Type II methanotrophs in each culture under initial condition (0 h in Cycle 1) was 1.7 × 10^4^ copies/µL, 1.4 × 10^4^ copies/µL, and 5.1 × 10^3^ copies/µL for OBAS (1:1, and 1:4) cultures and AS-only cultures (Fig. S1 and Fig. S2). The 16 S rRNA gene copy concentration of Type II methanotrophs increased gradually from 2.1 × 10^4^ copies/µL to 3.5 × 10^4^ copies/µL in OBAS (1:1) cultures (Fig. 4A). Importantly, the Type II methanotrophs to total bacteria ratio in Cycle 12 (44%) was twice as high as in Cycle 1 (22%), and 3.4 times higher than the initial condition (13%) (refer to Fig. 4A and Fig. S2). This result aligns with the PHB production discussed in Sect. 3.2. For OBAS (1:4) cultures, the 16S rRNA gene copy concentration of Type II methanotrophs in OBAS (1:4) cultures remained in the range between 1.2 × 10^4^ copies/µL and 1.3 × 10^3^ copies/µL, notably slightly higher in Cycle 4 and Cycle 12. However, the Type II methanotrophs to total bacteria ratio exhibited notable differences, showing 20% at the beginning, decreasing to 5%, and then increasing to 16% (Fig. 4). In contrast, AS-only culture showed that the proportion of Type II methanotrophs to total bacteria was approximately 1%. This outcome explains why AS-only culture accumulated a lower PHB content. In the OBAS (1:1) cultures, specifically, the relatively higher initial abundance of *M. trichosporium* OB3b expedited the establishment of conditions conducive to the prevalence of Type II methanotrophs. Conversely, in the 1:4 ratio condition, this establishment for favoring the dominance of PHB-accumulating methanotrophs was delayed. This resulted in an initial decline in Type II methanotrophs in Cycle 4, followed by a subsequent increase. Consequently, the initial *M. trichosporium* OB3b inoculation could have significantly influenced the establishment period of microbial community composition and targeted community composition, which can synthesize and utilize PHB.

The most intriguing observations were made in the *pmoA* gene copy concentration of *M. trichosporium* OB3b. The *pmoA* gene copy concentrations of *M. trichosporium* OB3b in cycle 1 were 2.9 × 10^4^ copies/µL and 1.2 × 10^4^ copies/µL in OBAS (1:1, and 1:4), respectively. The *M. trichosporium* OB3b to total bacteria ratio in Cycle 1 were 30% and 20%, respectively, identical to the results of Type II methanotrophs gene copy concentration. Under the initial conditions (0 h in Cycle 1), the *M. trichosporium* OB3b to total bacteria in OBAS (1:1, and 1:4) were 13%, and 5%, respectively (Fig. S2). This implies that bioaugmentation of *M. trichosporium* OB3b occurred in the first cycle. On the contrary, the *M. trichosporium* gene copy concentrations of AS-only cultures were below the detection limit (100 copies/µL), indicating the absence of *M. trichosporium* OB3b. Both OBAS cultures (1:1, and 1:4) decreased to 16 copies/µL and 384 copies/µL after cycle 1, respectively, whereas the copy numbers of OBAS (1:1) were below the detection limit. The percentages of *M. trichosporium* OB3b were almost 0% in all cultures after Cycle 1. Increased gene copy concentrations of Type II methanotrophs were anticipated for the successful bioaugmentation of *M. trichosporium* OB3b. However, the results of *M. trichosporium* OB3b gene copy concentration suggest that the effective bioaugmentation of *M. trichosporium* OB3b was only achieved after Cycle 1, rather than at the conclusion of Cycle 12. *M. trichosporium* OB3b-amended cultures might be dominated by other Type II methanotrophs (e.g.,* Methylocystis*, *Methylocella*, and *Methylocapsa*).

### Microbial community analysis

**Fig. 5:**
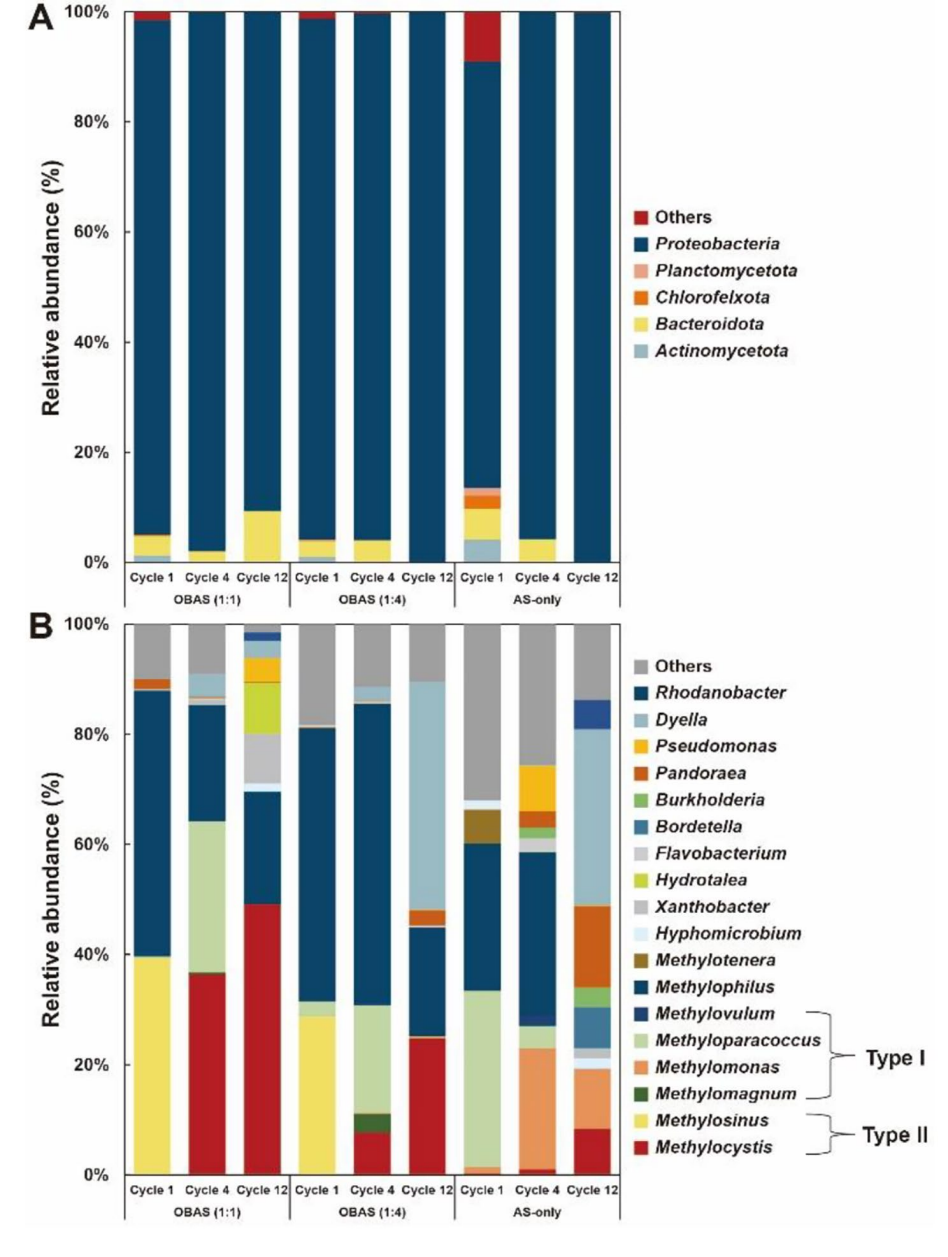
Taxonomy relative abundance of OBAS and AS-only cultures at the phylum level (**A**), and genus level (**B**). Relative abundances below 1% are classified as ‘Others’

To qualitatively analyze microbial compositions, 16S rRNA amplicon sequencing was conducted. At the phylum level, Proteobacteria dominated, accounting for over 70% of OBAS cultures and activated sludge. Notably, the genera *Methylocystis* and *Methylosinus* emerged as representative PHB-accumulating methanotrophs. In OBAS cultures (1:1, and 1:4), the *Methylosinus* genus accounted for 39.39% and 28.75%, respectively, with higher prevalence in the initial stages. However, across repeating cycles, the *Methylosinus* genus’s relative abundance declined, giving way to the *Methylocystis* genus (Fig. 5). Post-bioaugmentation with *M. trichosporium* OB3b, the *Methylocystis* genus dominated at 49.17% in OBAS (1:1) cultures, the highest observed across all cultures. This observation correlates with the highest PHB contents noted in OBAS cultures.

In the OBAS (1:1) culture, the proportion of Type II methanotrophs initially starts at 39.68% in Cycle 1 and later increases to 49.17% by Cycle 12. Concurrently, Type I methanotrophs, initially present in Cycle 1, became undetectable as cycles progressed. Likewise, the OBAS (1:4) culture exhibited Type II methanotrophs constituting 25.13% of the population by Cycle 12, with Type I methanotrophs also undetectable at the conclusion of the observation period (Fig. 5, Fig. S3, and Table 1). The invasion of Type I methanotrophs was not observed in the last cycle of OBAS cultures.

The consistent observations across both cultures imply a progressive increase in Type II methanotrophs coupled with a declining presence of Type I methanotrophs throughout repeated cycles. The stable maintenance of the microbial community structure corresponds with the predominance of Type II methanotrophs (Fig. 5 and Fig. S3). This indicates the successful establishment of a Type II methanotrophs-dominated culture. In contrast, AS-only cultures were dominated by Type I methanotrophs, constituting 33.11% (Cycle 1), 29.05% (Cycle 4), and 10.87% (Cycle 12) (Table 1) This outcome aligns with previous studies where Type I methanotrophs often predominate over Type II methanotrophs due to their higher affinity for methane [28, 30, 44]. *Methyloparacoccus* and *Methylomonas* were the most abundant in AS-only cultures (Fig. 5, and Table 2).

The relative abundance of Type II methanotrophs in AS-only cultures remained below 10%, suggesting that Type II methanotrophs require extended adaptation periods for their enrichment. From the perspective of methylotrophs, *Methylophilus*, a non-methanotrophic methylotroph capable of utilizing methanol, was frequently observed in methanotrophic enrichments in previous studies [33, 45–49]. Methanotrophic enrichments coexisting with methylotrophs and heterotrophs can eliminate toxic metabolites (e.g., methanol and formate) from methanotrophs’ methane oxidation. Additionally, *Methylocystis*- and *Methylophilus*-dominated cultures accumulated up to 59.4% of PHB during a more extended period of methane adaptation compared to this study [33].

Taxonomic classification and relative abundance of methanotrophs and methylotrophs are summarized (Tables 1 and 2). Importantly, the addition of *M. trichosporium* OB3b led to the outcompetition of Type I methanotrophs and successful enrichment into Type II methanotrophs-dominated cultures. Although initially *M. trichosporium* OB3b was dominant, *Methylocystis* spp. eventually became prevalent due to their versatility in oxygen and methane requirements. *Methylocystis* spp. is particularly robust under low methane conditions [50–52]. This is related to particulate methane monooxygenase A, which is the key enzyme of methane oxidation. In our batch feeding cycle, the batch supply of methane and oxygen, may be a critical factor favoring the growth of these methanotrophs after the methane consumption of *M. trichosporium* OB3b. Previous studies reported that nitrogen selection (i.e., ammonium inhibition), cyclic methane, and nitrogen starvation were involved in securing Type II methanotrophic enrichments, especially *Methylocystis*-dominated cultures [29, 30, 36, 53]. However, in our research, the bioaugmentation of *M. trichosporium* might play a vital role for enriching PHB-accumulating methanotrophic enrichments.

**Table 1:** Relative abundance of methanotrophs. All values are presented in percentage (%)

Genus	OBAS (1:1)			OBAS (1:4)			AS-only		
	Cycle 1	Cycle 4	Cycle 12	Cycle 1	Cycle 4	Cycle 12	Cycle 1	Cycle 4	Cycle 12
*Methylosinus*	39.39	0.00	0.00	28.68	0.00	0.30	0.00	0.00	0.00
*Methylocystis*	0.04	36.46	49.17	0.07	7.72	24.83	0.31	1.03	8.38
**Type II** **methanotrophs**	**39.43**	**36.46**	**49.17**	**28.75**	**7.72**	**25.13**	**0.31**	**1.03**	**8.38**
*Methylomagnum*	0.00	0.29	0.00	0.00	3.34	0.00	0.00	0.00	0.00
*Methylomonas*	0.00	0.00	0.00	0.02	0.22	0.00	1.14	21.95	10.87
*Methyloparacoccus*	0.25	27.46	0.00	2.74	19.55	0.00	31.97	4.12	0.00
*Methylovulum*	0.00	0.00	0.00	0.04	0.44	0.00	0.00	1.95	0.00
**Type I methanotrophs**	**0.25**	**27.75**	**0.00**	**2.79**	**23.56**	**0.00**	**33.11**	**28.02**	**10.87**
**Total**	**39.68**	**64.21**	**49.17**	**31.55**	**31.28**	**25.13**	**33.43**	**29.05**	**19.25**

**Table 2:** Relative abundance of methylotrophs. All values are presented in percentage (%)

Genus	OBAS (1:1)			OBAS (1:1)			AS-only		
	Cycle 1	Cycle 4	Cycle 12	Cycle 1	Cycle 4	Cycle 12	Cycle 1	Cycle 4	Cycle 12
*Methylophilus*	48.21	21.09	20.42	49.59	54.29	19.87	26.75	29.51	0.01
*Methylotenera*	0.02	0.00	0.00	0.23	0.08	0.00	6.17	0.05	0.00
*Hyphomicrobium*	0.06	0.03	1.49	0.01	0.02	0.07	1.63	0.07	1.97
*Xanthobacter*	0.00	0.93	8.99	0.03	0.15	0.17	0.00	0.00	1.81
**Total**	**48.28**	**22.05**	**30.90**	**49.86**	**54.53**	**20.11**	**34.54**	**29.63**	**3.79**

## Conclusion

This study investigated the impact of incorporating pure methanotrophs into activated sludge, examining methane oxidation, PHB production, gene copy concentrations, and microbial community dynamics. Quantitative PHB accumulation tests demonstrated that only activated sludge bioaugmented with *M. trichosporium* OB3b has the capability of PHB production. Maximum PHB contents and concentration of 37% and 96 mg/L, respectively, were observed, significantly surpassing those in activated sludge without *M. trichosporium* OB3b. Quantification of *M. trichosporium* OB3b and Type II methanotrophs unveiled that the bioaugmentation of *M. trichosporium* OB3b occurred in the initial stages, triggering the involvement of other Type II methanotroph for efficient PHB production. Microbial community analysis suggested that *Methylocystis*-dominated cultures played a pivotal role in synthesizing PHB. To validate microbial shifts in activated sludge with *M. trichosporium* OB3b, metabolite analysis is recommended to confirm the influences of *M. trichosporium* OB3b in activated sludge. This approach could be an effective strategy to enrich Type II methanotrophs-dominated culture by bioaugmentation with *M. trichosporium* OB3b in an activated sludge system for enhancing PHB production and rapidly securing the PHB-accumulating methanotrophic enrichments.

### Electronic supplementary material

Below is the link to the electronic supplementary material.

Supplementary Material 1

## Data Availability

No datasets were generated or analysed during the current study.
