# Supplementary material for: Methylosinus trichosporium OB3b bioaugmentation unleashes polyhydroxybutyrate-accumulating potential in waste-activated sludge
Source: Microb Cell Fact. 2024 May 31;23:160. doi: 10.1186/s12934-024-02442-w (PMC11140957; doi:10.1186/s12934-024-02442-w)
Supplement: Supplementary file 1 — Supplementary Material 1 [file 12934_2024_2442_MOESM1_ESM.docx]

**Supplementary material**

***Methylosinus trichosporium* OB3b bioaugmentation unleashes polyhydroxybutyrate-accumulating potential in waste-activated sludge**

**Hyerim Eam^1^, Dayoung Ko^2^, Changsoo Lee^2^, Jaewook Myung^1^***

^1^ Department of Civil and Environmental Engineering, KAIST, Daejeon 34141, Republic of Korea

^2^Department of Civil, Urban, Earth, and Environmental Engineering, UNIST, Ulsan 44919, Republic of Korea

***Corresponding author. Mailing address:** [**jjaimyung@kaist.ac.kr**](mailto:jjaimyung@kaist.ac.kr)

| **Primer** | **Sequences (5’ to 3’)** | **Target** | **Reference** |
| --- | --- | --- | --- |
| 338F | ACTCCTRCGGGAGGCAGCAG | Universal bacteria  (16S rRNA gene) | [1, 2] |
| 806R | GGACTACNVGGGTWTCTAAT |  |  |
| 27F | GAGAGTTTGATCMTGGCTCAG | Type II methanotrophs  (16S rRNA gene) | [3, 4] |
| MethT2R | CATCTCTGRCSAYCATACCGG |  |  |
| OB3B_pmoAF | TTCTGGGGCTGGACCTAYTTC | *M. trichosporium* OB3b (*pmoA* gene) | [5, 6] |
| OB3B_pmoAR | CCGACAGCAGCAGGATGATG |  |  |

**Table S1.** Specific PCR primers used in this study.

**References:**

[1] Yu Y, Lee C, Kim J, Hwang S. Group-specific primer and probe sets to detect methanogenic communities using quantitative real-time polymerase chain reaction. Biotechnol. Bioeng. 2005;89(6):670-9; doi: 10.1002/bit.20347.

[2] Elwood HJ, Olsen GJ, Sogin ML. The Small-Subunit Ribosomal-Rna Gene-Sequences from the Hypotrichous Ciliates Oxytricha-Nova and Stylonychia-Pustulata. Mol. Biol. Evol. 1985;2(5):399-410.; doi: 10.1093/oxfordjournals.molbev.a040362

[3] Wise MG, McArthur JV, Shimkets LJ. Methanotroph diversity in landfill soil: Isolation of novel type I and type II methanotrophs whose presence was suggested by culture-independent 16S ribosomal DNA analysis. Appl. Environ. Microbiol. 1999;65(11):4887-97.

[4] McDonald IR, Bodrossy L, Chen Y, Murrell JC. Molecular ecology techniques for the study of aerobic methanotrophs. Appl. Environ. Microbiol. 2008;74(5):1305-15; doi: 10.1128/Aem.02233-07.

[5] Tentori EF, Richardson RE. Methane Monooxygenase Gene Transcripts as Quantitative Biomarkers of Methanotrophic Activity in OB3b. Appl. Environ. Microbiol. 2020;86(23); doi: 10.1128/AEM.01048-20.

[6] Knapp CW, Fowle DA, Kulczycki E, Roberts JA, Graham DW. Methane monooxygenase gene expression mediated by methanobactin in the presence of mineral copper sources. Proc. Natl. Acad. Sci. U.S.A. 2007;104(29):12040-5; doi: 10.1073/pnas.0702879104.


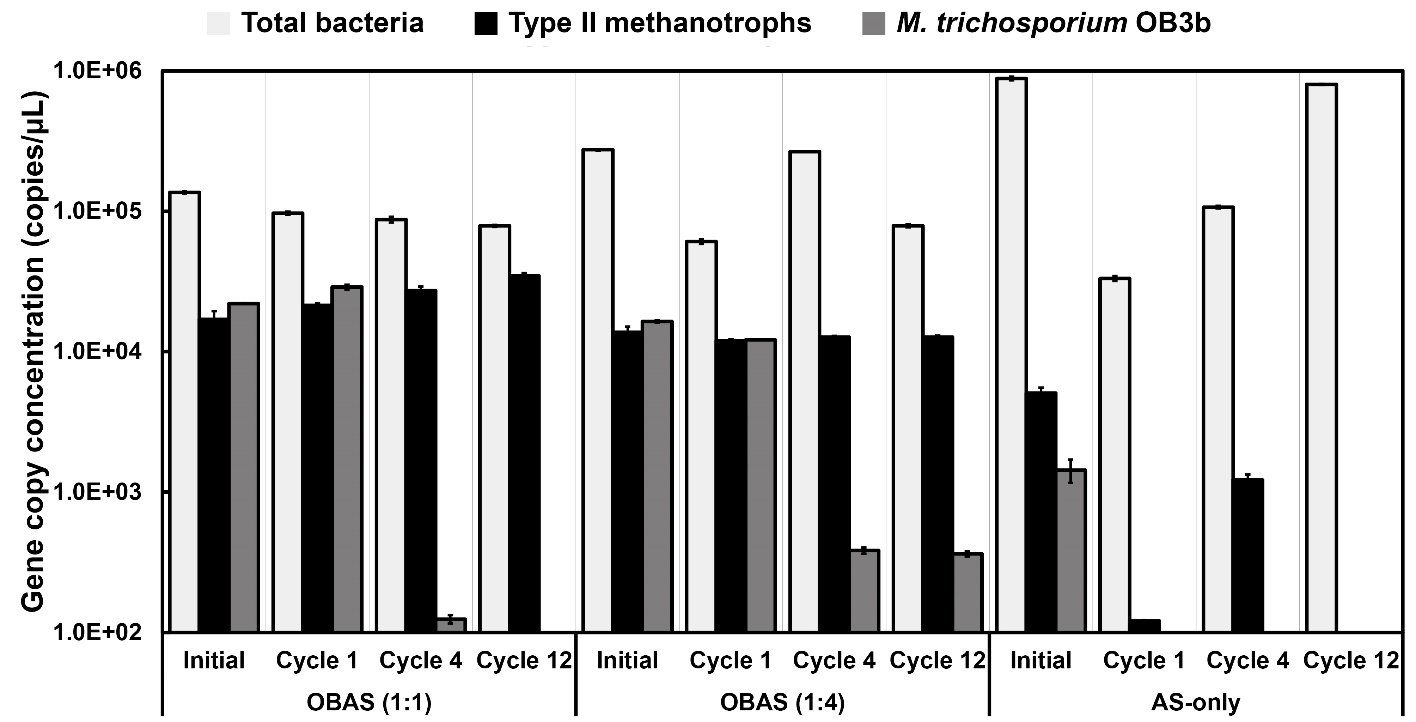


**Fig. S1.** The gene copy concentration of total bacteria, Type II methanotrophs, and *M. trichosporium* OB3b. The cultures from the initial condition were measured at 0 h in Cycle 1. Each culture, denoted with cycle numbers was analyzed at 72 h in each cycle.

**
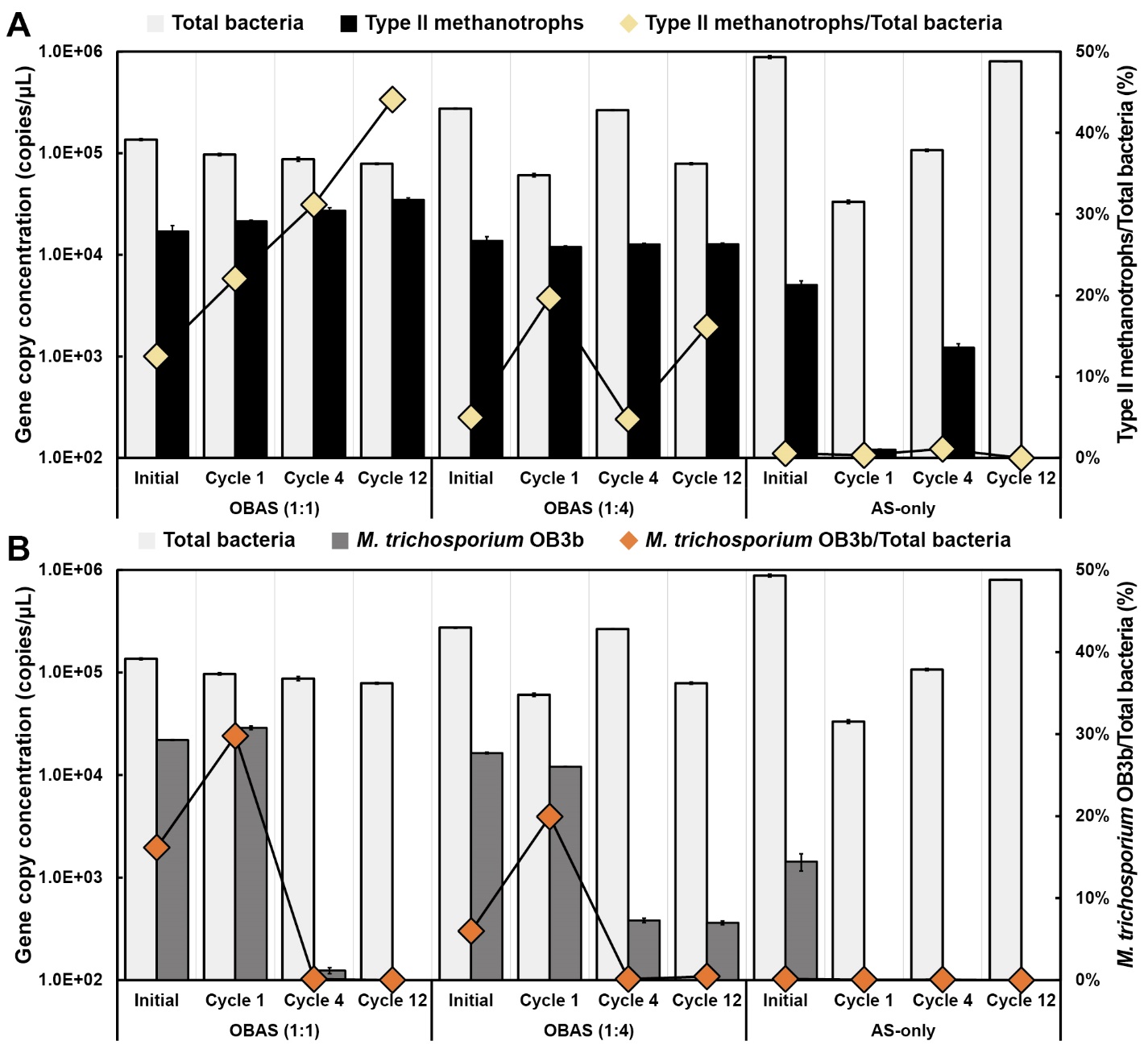
**

**Fig. S2.** The gene copy concentrations of total bacteria, Type II methanotrophs (A), and *M. trichosporium* OB3b (B) in OBAS and AS-only cultures. The ratio of type II methanotrophs (A) and *M. trichosporium* OB3b (B) to total bacteria were monitored. The cultures from the initial condition were measured at 0 h in Cycle 1. Each culture, denoted with cycle numbers was analyzed at 72 h in each cycle. Below 100 copies/μl were set as the limit of detection (LOD).


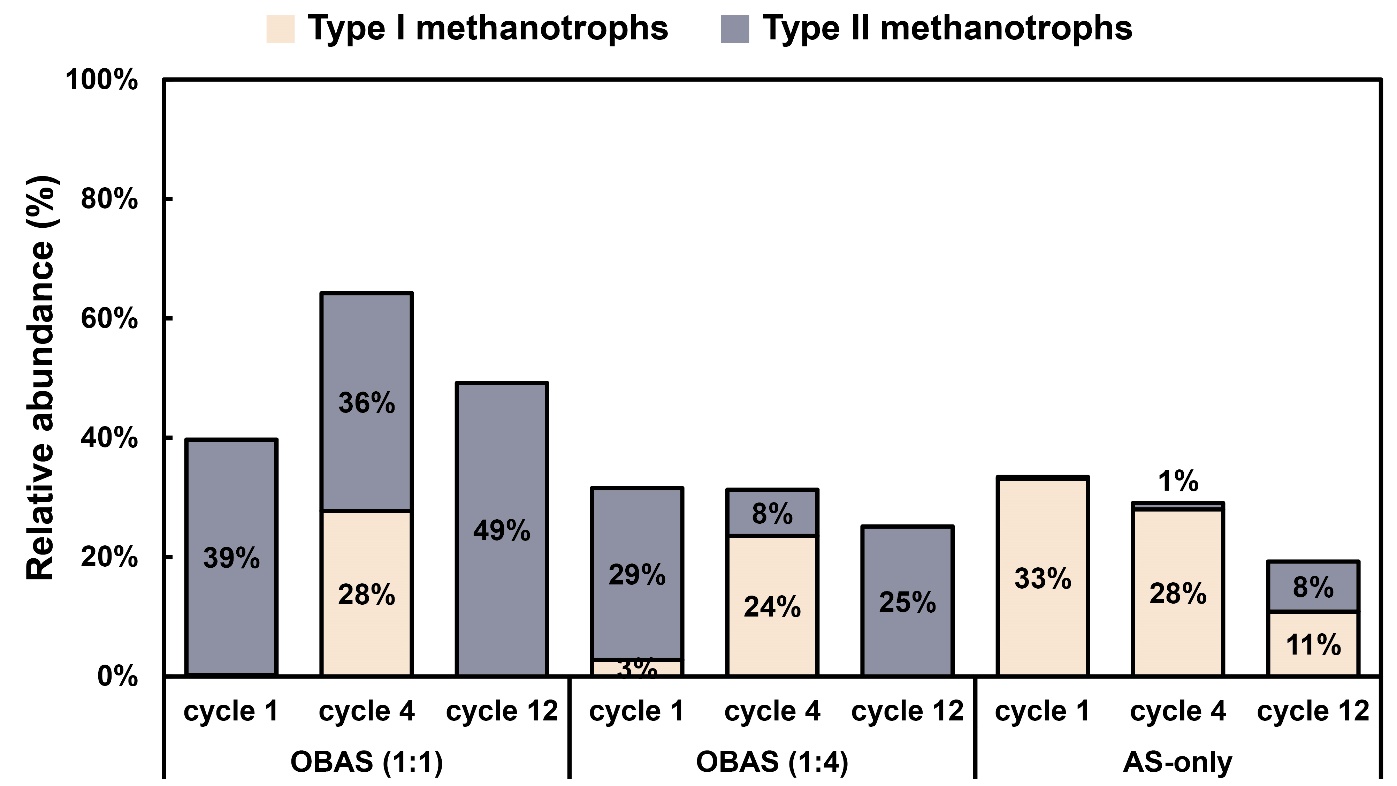


**Fig. S3.** Relative abundance of Type I and Type II methanotrophs during repeating cycles under different conditions (OBAS (1:1), OBAS (1:4), and AS-only cultures).
